# Characterization of lactylation-related genes for predictive model development and molecular subtyping in HBV-associated hepatocellular carcinoma

**DOI:** 10.1515/biol-2025-1394

**Published:** 2026-11-06

**Authors:** Jinlian Li, Xipeng Yan, Xinwei Wang, He Xie, Jujun Sun, Qunying Wu, Limin Chen

**Affiliations:** The Joint Laboratory on Transfusion-Transmitted Diseases (TTDs) Between Institute of Blood Transfusion, Chinese Academy of Medical Sciences and Nanning Blood Center, Nanning Blood Center, Nanning, China; The Hospital of Xidian Group, Xi’an, China; School of Intelligent Medicine and Biotechnology, Guilin Medical University, Guilin, China; Institute of Blood Transfusion, Chinese Academy of Medical Sciences and Peking Union Medical College, Chengdu, China

**Keywords:** HBV-HCC, machine learning, diagnostic biomarkers, lactylation-related genes, predictive model

## Abstract

Chronic hepatitis B virus (HBV) infection is the leading risk factor for hepatocellular carcinoma (HCC). Lactylation-related genes (LRGs) participate in HCC progression, yet their roles in HBV-associated HCC (HBV-HCC) remain largely unclear. In this study, we screened differentially expressed LRGs (DE-LRGs) from HBV-HCC transcriptomic data using weighted gene co-expression network analysis (WGCNA) and built a high-precision random forest prediction model. Three hub genes (RACGAP1, STMN1, ILF2) were verified to be overexpressed in HBV-HCC tissues across multiple datasets and quantitative real-time polymerase chain reaction (qPCR), and their high expression correlated with unfavorable overall survival and distinct immune infiltration. A three-gene nomogram was constructed in the GSE47197 cohort, with an area under the curve (AUC) of 0.928 validated by calibration curves, decision curve analysis (DCA) and receiver operating characteristic (ROC) curves. External validation in GSE121248 reached an AUC of 0.965, verifying its robust predictive power. Moreover, consensus clustering divided HBV-HCC into two LRG-based molecular subtypes, where Cluster 2 had worse prognosis. Collectively, this study establishes an LRG-based predictive model and identifies two molecular subtypes for HBV-HCC, providing reliable and promising biomarker candidates for HBV-HCC risk prediction and individualized immunotherapy.

## 1 Introduction

Chronic hepatitis B virus (HBV) infection is a major risk factor for liver cirrhosis and hepatocellular carcinoma (HCC), contributing substantially to global liver-related mortality [1]. Epidemiologically, approximately 248 million individuals worldwide suffer from chronic HBV infection, causing more than 600,000 annual deaths from HBV-associated complications [2]. The prevalence of HBV infection is tightly correlated with the incidence and mortality of HCC globally [3]. Clinically, HBV infection accounts for over half of HCC cases and nearly one-third of HCC-related deaths worldwide [4], 5]. Integration of HBV gene fragments into the host genome is recognized as a core mechanism for the development of HBV-associated HCC (HBV-HCC) [6]. This integration disrupts the expression of host growth regulatory genes, oncogenes, and tumor suppressor genes (e.g., TERT), thereby driving malignant transformation and tumor progression [6], 7]. Moreover, persistent chronic inflammation induced by chronic HBV infection further promotes HCC occurrence and distant metastasis [8]. Due to the atypical and asymptomatic features of early-stage HBV-HCC, most patients are diagnosed at advanced stages with limited treatment options, resulting in poor overall survival [9]. Therefore, it is essential to identify novel and reliable biomarkers for the early identification and prognostic evaluation of HBV-HCC to improve clinical outcomes.

Lactic acid, a classic glycolytic metabolite of the Warburg effect, was previously considered merely a metabolic end product [10]. However, emerging evidence has confirmed that lactic acid promotes tumor cell proliferation, survival, and angiogenesis via lactate dehydrogenase (LDH) and monocarboxylic acid transporters (MCTs), ultimately fostering an immunosuppressive tumor microenvironment [11]. As a newly identified lactate-dependent post-translational modification (PTMs), protein lactylation has been proven to exert crucial regulatory functions in multiple biological and pathological processes independent of glycolytic metabolism [12]. Accumulating studies have demonstrated that aberrant lactylation modification is closely linked to HCC occurrence and progression [13]. Specifically, intracellular lactate-derived protein lysine lactylation (Kla) promotes the malignant phenotype of HCC cells [14]. Elevated histone lactylation levels (H3K9la and H3K56la) in HCC tissues are significantly correlated with adverse clinical prognosis [15]. Dysregulated lactylation of functional proteins such as IGF2BP3 also mediates therapeutic resistance in HCC [16]. In addition, hepatic stellate cells have been shown to stimulate HCC progression by modulating histone lactylation status [17]. Previous studies have also reported upregulated expression of lactylation-modifying enzymes (EP300, HDAC1–3) and increased overall lactylation levels in HCC tissues compared to normal liver tissues [18]. Furthermore, lactylation-related signatures have shown promising predictive efficacy for HCC prognosis and therapeutic response [19]. Despite these advances, the clinical value and biological roles of lactylation-related genes (LRGs) in HBV-HCC remain insufficiently elucidated. The potential of LRGs for prognostic prediction, immune microenvironment regulation, and molecular subtyping in HBV-HCC has not been systematically explored, leaving a critical research gap in this field.

In recent years, various computational and machine-learning algorithms have been developed to facilitate biological data analysis and post-translational modification research. These methodological approaches cover gene regulatory network reconstruction, high-dimensional biological feature screening, and protein modification site prediction [20], [21], [22], [23]. These studies demonstrate the feasibility of feature-oriented computational paradigms for dissecting complex biological datasets. Nevertheless, most of these studies focus primarily on algorithm optimization and methodological exploration, rather than constructing disease-specific predictive signatures for HBV-HCC.

To address the above research gap, we systematically explored LRGs in HBV-HCC using transcriptomic data from the gene expression omnibus (GEO) database. Integrating differential expression analysis and weighted gene co-expression network analysis (WGCNA), we identified differentially expressed LRGs (DE-LRGs) by comparing HBV-infected non-tumorous liver tissues and HBV-HCC tumor tissues. Multiple machine learning models were constructed based on DE-LRGs, and the hub genes identified from the optimal model were validated in independent GEO and TCGA cohorts as well as via quantitative real-time polymerase chain reaction (qPCR) experiments. We comprehensively evaluated the prognostic value, predictive efficacy, and clinical applicability of the LRG-based signature through survival analysis, receiver operating characteristic (ROC) curves, decision curve analysis (DCA), and nomogram validation. Finally, unsupervised consensus clustering was performed to stratify LRG-based molecular subtyping of HBV-HCC patients. The study workflow is outlined in Figure 1.

**Figure 1:**
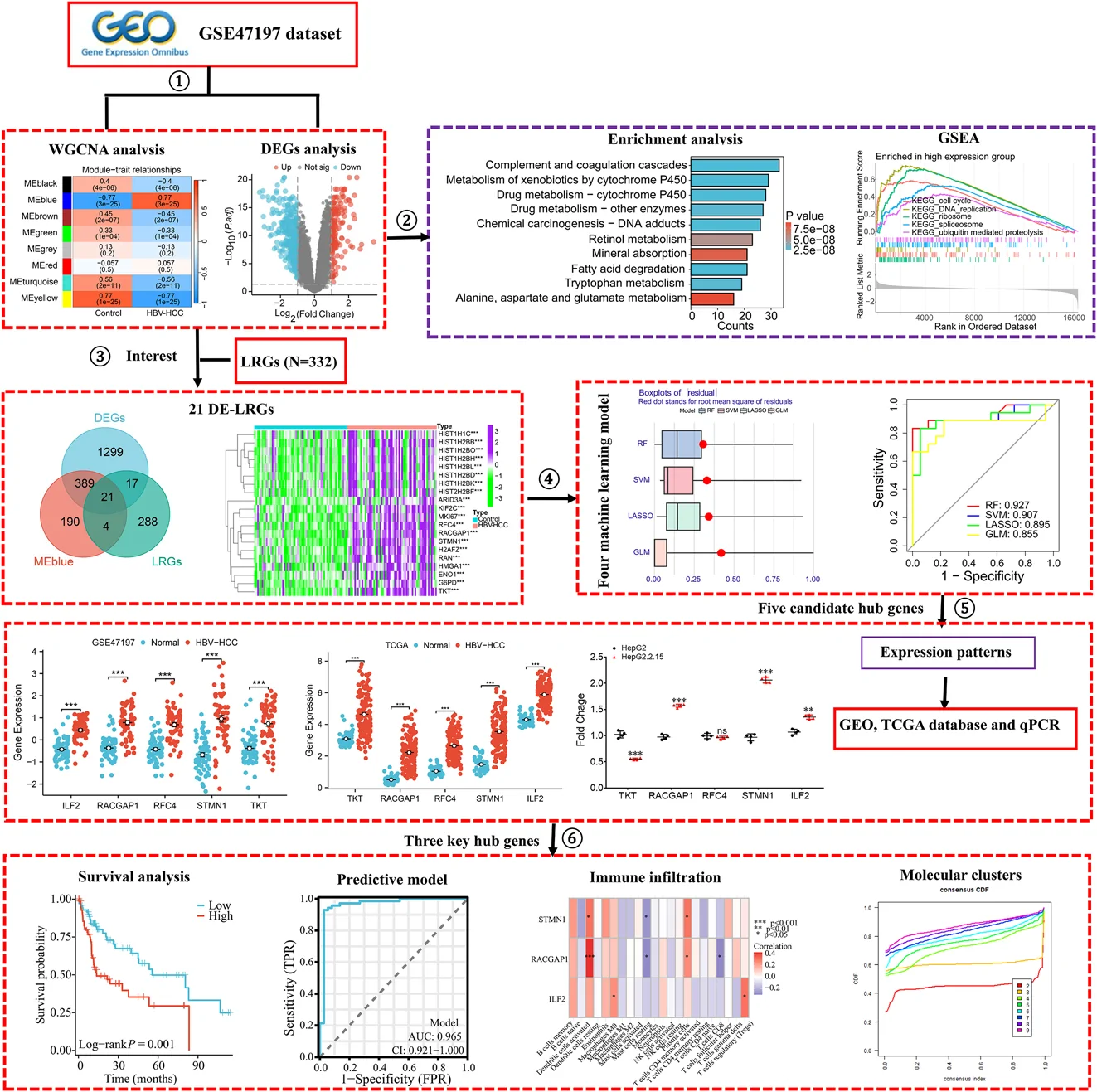
Outline of the flowchart of this study.

## 2 Materials and methods

### 2.1 Data collection and preparation

The raw gene expression profile datasets were retrieved from the GEO database. All dataset contains both adjacent normal liver tissue from HBV-infected patients and HBV-HCC tumor tissue to specifically model HBV-driven hepatocarcinogenesis. Detailed information on each dataset is summarized in Supplementary Table S1. Specifically, GSE47197 was designated as the training cohort, consisting of 63 adjacent normal liver tissues from HBV patients and 61 HBV-HCC tumor specimens. GSE121248, comprising 37 adjacent normal liver tissues and 70 HBV-HCC tumor specimens,was served as the independent external validation cohorts. The initial probe-level expression matrix was transformed into gene-level expression matrix using Strawberry Perl (version 5.30.0.1) with corresponding platform annotation files. For subsequent data processing procedure, expression normalization was performed using the “preprocessCore” package. Principal component analysis (PCA) was further conducted to verify the homogeneity of normalized transcriptomic data. A total of 332 LRGs were obtained from a keyword-based search for “lactate” from the molecular signatures database (MsigDB, https://www.gsea-msigdb.org/gsea/msig db/) [19]. The complete list of these 332 LRGs is provided in Supplementary Table S2.

### 2.2 Identification of differentially expressed genes (DEGs)

The “limma” package was used to screen differentially expressed genes (DEGs) between HBV-infected non-tumorous liver tissues and HBV-HCC tumour tissues within the training dataset GSE47197. DEGs were identified by the thresholds |log2 (fold change, FC)|>0.585 and an adjusted *P* < 0.05. Volcano plots visualizing the distribution of DEGs according to fold change and statistical significance were generated via the “ggplot2” package. In addition, the expression patterns of the top 50 DEGs across all samples were displayed in heatmaps constructed using the “ComplexHeatmap” package.

### 2.3 Functional annotation analysis

We employed the “ClusterProfiler” package to perform gene ontology (GO) and kyoto encyclopedia of genes and genomes (KEGG) enrichment analyses to better understand the biological functions of DEGs. The GO framework includes three main ontologies: molecular function (MF), cellular component (CC), and biological process (BP). We further performed gene set enrichment analysis (GSEA) against all genes in the training cohort to compare pathway alterations between high-expression and low-expression subgroups using the “clusterProfiler” package. The reference gene set “c2.cp.kegg.symbols.gmt” was retrieved from the MSigDB. Gene sets with normalized enrichment scores (NES) were ranked, and the top five enriched gene sets from both upregulated and downregulated groups were displayed. Enrichment results with *P* < 0.05 were visualized using “ggplot2”.

### 2.4 WGCNA

WGCNA was performed on the GSE47197 training dataset to detect gene modules closely correlated with HBV-HCC phenotypes. Initially, the “goodSamplesGenes” function was employed to filter out genes and samples with excessive missing values for data quality control. The “pickSoftThreshold” function was then utilized to determine the optimal soft thresholding power (*β*), which was adopted to construct the topological overlap matrix (TOM). Gene modules with similar expression trends were identified by dynamic tree cutting. Gene significance (GS) and module membership (MM) were calculated to quantify correlations between genes and clinical traits, and genes within key modules were extracted for subsequent analysis. Venn diagrams were generated using the “ggvenn” package to intersect DEGs, key module genes and LRGs to obtain DE-LRGs. The expression distribution of DE-LRGs was visualized by box plots and heatmaps using “ggpubr” and “pheatmap”. Moreover, GO and KEGG enrichment analyses of DE-LRGs were carried out with the “clusterProfiler” package to explore their potential biological functions.

### 2.5 Optimization of the prediction model

Four machine learning algorithms, including random forest (RF), support vector machine (SVM), generalized linear model (GLM) and least absolute shrinkage and selection operator (LASSO), were applied to build predictive models based on the expression matrix of 21 DE-LRGs [24], [25], [26], [27]. Model construction and optimization were implemented via the “caret” package, and these algorithms have been widely validated for biomarker screening in previous studies [28], 29]. To ensure analytical reproducibility, a fixed random seed (set.seed(123)) was applied throughout all modelling procedures. The whole dataset was randomly divided into a training set (70 %) and a test set (30 %) using the “createDataPartition” function. Five-fold repeated cross-validation was adopted during model training to mitigate overfitting and improve generalization performance. The “DALEX” package was used to generate model explainers; a customized probability prediction function was defined to output classification probabilities, and multiple evaluation metrics were calculated systematically. Reverse cumulative distribution curves and boxplots of residuals were plotted to assess model fitting. Receiver operating characteristic (ROC) curves and corresponding area under the curve (AUC) values were computed using the “pROC” package to quantify the diagnostic performance of each model. Variable importance analysis was further conducted to evaluate the contribution of each DE-LRGs. The optimal predictive model was selected comprehensively according to AUC, stability, residual distribution and interpretability. The top five genes with the highest variable importance scores in the optimal model were recognized as hub genes for subsequent analyses.

### 2.6 Expression pattern analysis of hub genes

Transcriptomic data from GEO datasets (GSE47197 and GSE121248) were first used to verify the expression of hub genes; the ggplot2 package was adopted to compare gene expression levels between HBV-HCC patients and HBV-infected non-tumorous control samples. Subsequently, transcriptomic and matched clinical data including 50 normal liver samples and 371 HCC samples were obtained from the cancer genome atlas (TCGA) database. Clinical metadata downloaded from university of California, Santa Cruz (UCSC) Xena were used to filter a subgroup of 145 patients with HBV-HCC for subsequent analysis. Raw gene expression values in fragments per kilobase of transcript per million mapped reads (FPKM) were log_2_-transformed. We compared the mRNA expression levels of hub genes in two sets: HCC versus normal tissues, and HBV-HCC versus normal tissues.

### 2.7 Cell culture

The human liver cancer cell lines HepG2 and HepG2.2.15 were provided from Institute of Blood Transfusion, Chinese Academy of Medical Sciences and Peking Union Medical College. Both HepG2 and HepG2.2.15 cells stably harboring the entire HBV genome with the ability to consistently produce infectious HBV particles, were maintained in minimum essential medium (MEM; Gibco, USA), supplemented with 10 % fetal bovine serum (FBS; AN Biotech, Germany) and 0.5 % mycoplasma removal agent. The cultures were incubated at 37 °C in 5 % CO2 incubator. To maintain selective pressure, the HepG2.2.15 culture medium was supplemented with 400 μg/mL G418.

### 2.8 qPCR analysis

Total RNA was isolated from the cultured cells by Trizol reagent (Invitrogen, USA). Complementary deoxyribonucleic acid (cDNA) synthesis was performed using Toyobo’s (Japan) high-capacity cDNA Reverse Transcription Kit, following the manufacturer’s guidelines. qPCR analysis was carried out on a LightCycler 480 system (Roche, Switzerland) using the 2 × PCR Master Mix (Toyobo, Japan). The fold change of all target genes, normalized against GAPDH as an internal control, was computed using the 2^−ΔΔCt^ method. All primer sequences for qPCR, which were custom-designed and manufactured by Shenggong Biotech (Shanghai, China), are provided in Supplementary Table S3.

### 2.9 Prognostic evaluation of hub genes

The optimal expression cut-off value for hub gene was calculated using the “surv_cutpoint” function from the “survminer” R package, which stratified HBV-HCC or HCC patients into high-expression or low-expression subgroup. Kaplan–Meier survival analysis with log-rank tests was performed to evaluate differences in overall survival (OS) between subgroups. Statistical significance was set at *P* < 0.05.

### 2.10 Development and validation of nomogram models

Nomogram is a widely used tool for clinical outcome (prognosis) prediction, which converts complex statistical models into intuitive numerical scales to calculate the probability of clinical events [30]. Based on the expression of hub genes, a nomogram model for HBV-HCC was constructed using the “rms” package. Calibration curves, DCA curves, clinical impact curves, and ROC curves were applied to comprehensively assess the predictive performance of the nomogram. The independent GSE121248 cohort was used for external validation to evaluate model stability and predictive accuracy.

### 2.11 Immune infiltration analysis

To characterize the tumor immune microenvironment, we estimated the relative abundances of 22 immune cell types using the Cell-type Identification by Estimating Relative Subsets of RNA Transcripts (CIBERSORT) deconvolution algorithm, with its standard leukocyte gene signature matrix (LM22) [31]. Only samples with a CIBERSORT output *P* < 0.05 were considered to have reliable deconvolution results and were retained for downstream analysis. The estimated immune cell fractions were then compared between the HBV-HCC and control groups (GSE47197) using boxplots. Furthermore, Spearman correlation analysis was conducted to assess the relationships between expression levels of the diagnostic biomarkers and the inferred immune cells abundances. The “corrplot” package was utilized to visualize the results, with statistical significance set at *P* < 0.05.

### 2.12 Unsupervised clustering analysis of HBV-HCC patients

Unsupervised consensus clustering was performed using the “ConsensusClusterPlus” package to stratify 145 HBV-HCC patients from the TCGA cohort into molecular subtypes based on the expression profiles of the five hub genes. The parameters were set as follows: maximum cluster number (maxK = 9), 50 resampling iterations (reps = 50), 80 % sample resampling (pItem = 0.8), and Euclidean distance. The optimal cluster number (*k* = 2) was determined by the CDF curve, consensus matrix, and relative changes in the area under the CDF curve. Patients were classified into Cluster 1 (high-risk subtype) and Cluster 2 (low-risk subtype). Moreover, PCA was performed to validate the distribution of the two clusters. Kaplan-Meier survival analysis was conducted using the “survival” and “survminer” packages to compare prognosis between the two clusters. Gene expression heatmaps were generated with the “pheatmap” package, and the Wilcoxon rank-sum test was applied to evaluate differences in hub gene expression levels between clusters.

### 2.13 Statistical analysis

All analyses of results were performed using GraphPad Prism 6.0, where a two-tailed Student’s *t*-test was applied for comparative evaluations. To explore the relationships among variables, Spearson correlation analysis was utilized. Each experiment was repeated in three independent biological replicates, and the results are expressed as mean ± SEM. *P* < 0.05 was considered statistically significant.

## 3 Results

### 3.1 Identification of DEGs from HBV-HCC and functional enrichment analysis

A total of 611 DEGs between HBV and HBV-HCC were identified, as depicted in the volcano plots (Figure 2A). To clearly visualize the most robust expression changes, we selected the top 50 most significantly DEGs for display in a heatmap (Figure 2B). The functional enrichment analysis revealed that these DEGs were primarily linked to different biological processes, including fatty acid metabolism, organic acid biosynthesis, carboxylic acid biosynthesis, as well as the catabolism of both organic and carboxylic acids (Figure 2C). KEGG pathway analysis highlighted that these DEGs were mainly associated with complement and coagulation cascades, cytochrome P450-mediated xenobiotic metabolism, drug metabolism involving cytochrome P450 and other enzymes, retinol metabolism, mineral absorption, and the degradation of fatty acids (Figure 2D). Additionally, GSEA indicated that the high expression group exhibited a strong correlation with pathways related to the cell cycle, DNA replication, ribosome function, spliceosome activity, and ubiquitin-mediated proteolysis (Figure 2E). In contrast, the low expression group demonstrated significant enrichment in pathways related to complement and coagulation cascades, cytochrome P450 drug metabolism, and fatty acid metabolism (Figure 2F).

**Figure 2:**
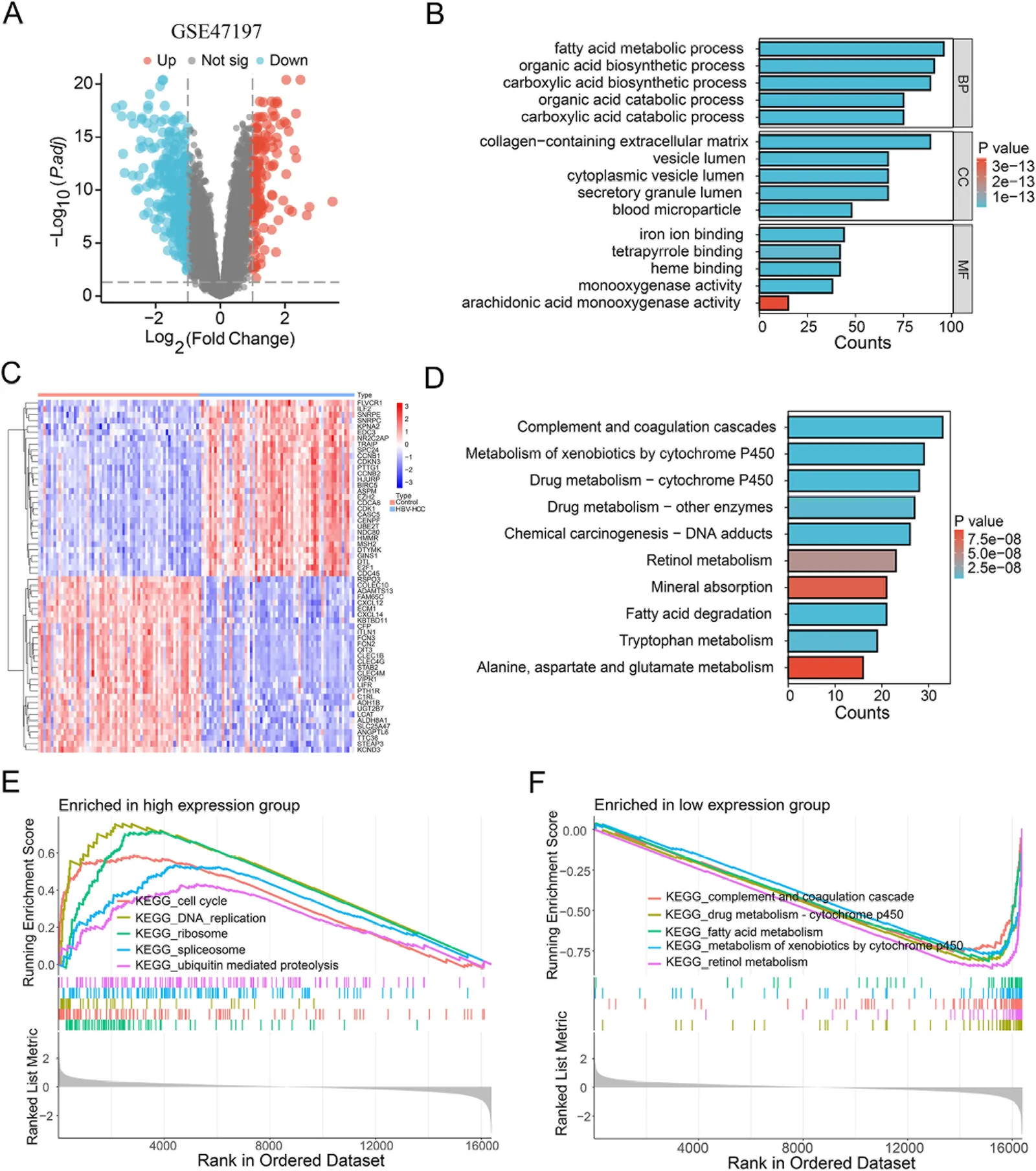
Identification of DEGs and functional enrichment analysis in HBV-HCC in GSE47197 cohort. (A) Volcano map of DEGs. (B) Heat map of DEGs. (C) GO enrichment analysis. (D) KEGG enrichment analysis. (E) GSEA enrichment analysis in the high expression group. (F) GSEA enrichment analysis in the low expression group.

### 3.2 Gene module screening and DE-LRGs selection

WGCNA was conducted utilizing the expression profiles from the GSE47197 dataset to identify critical gene modules linked to HBV-HCC. An *β* of 9 was determined based on its favorable scale independence and average connectivity (Figure 3A). The application of a hierarchical clustering algorithm facilitated the segmentation of the clustering dendrogram into eight distinct gene modules, each denoted by a unique color (Figure 3B). Subsequent clinical correlation analysis indicated that the Module Eigengene blue (MEblue) module displayed the most substantial positive correlation with HBV-HCC, comprising 604 genes and exhibiting a correlation coefficient of *r* = 0.77 (*P* = 3e - 25) (Figure 3C). The scatter plot demonstrated a robust correlation between GS and MM within the “MEblue” module, yielding a correlation coefficient of 0.91 (*P* < 1e - 200) (Figure 3D). Additionally, a Venn diagram elucidated the overlap, pinpointing 21 DE-LRGs that hold significant relevance in the context of HBV-HCC (Figure 3E). The heatmap depicted the distribution of these 21 DE-LRGs, all of which exhibited significant upregulation in HBV-HCC compared to HBV (Figure 3E and F). The functional enrichment analysis pertaining to the 21 DE-LRGs is presented in Figure S1.

**Figure 3:**
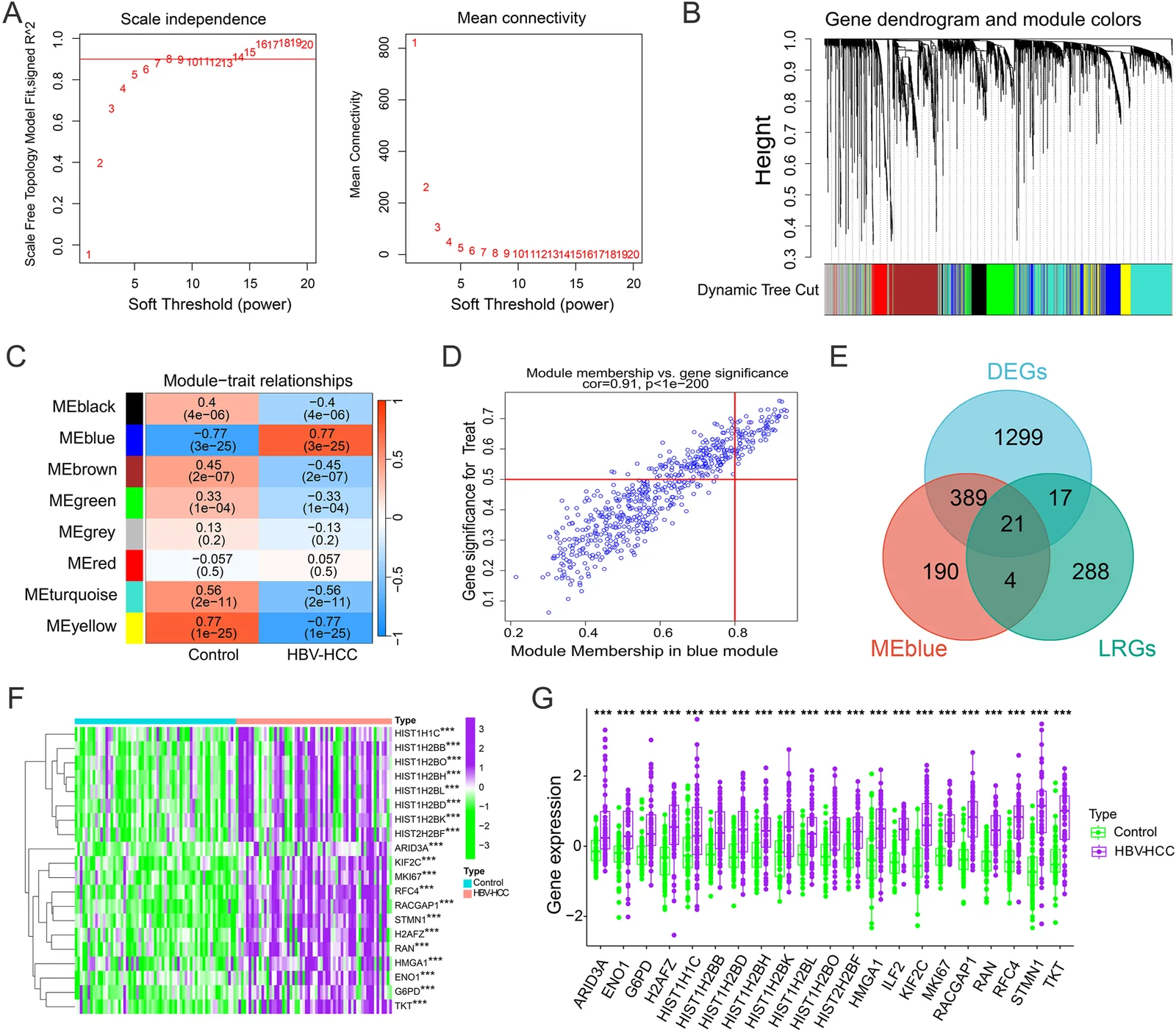
Identification of WGCNA network modules in HBV-HCC. (A) Scale-free topology analysis. (B) The gene dendrogram and module color are illustrated in WGCNA. (C) The correlation of each module’s eigengene with the phenotype. (D) Correlation scatter plots for GS and MM in the MEblue module. (E) Venn diagram for 21 DE-LRGs from DEGs, MEblue, and LRGs. (F) Heatmap of 21 DE-LRGs. (G) Expression patterns of 21 DE-LRGs.

### 3.3 Creation of the best prediction model

Four distinct machine learning models, namely RF, SVM, LASSO, and GLM, were established utilizing 21 DE-LRGs derived from the training cohort. Among these models, the RF approach showed the smallest residual values (Figure 4A and B). We then ranked the top 10 significant features for each model based on their root mean square error (RMSE), as shown in Figure 4C. The performance of the four algorithms in the training cohort was further evaluated using ROC curves. The RF model achieved the highest AUC of 0.927, in contrast to the AUC values of 0.907 for the SVM model, 0.895 for the LASSO model, and 0.855 for the GLM model (Figure 4D). Finally, we selected the five most influential variables from the RF model – TKT, RACGAP1, RFC4, STMN1, and ILF2 – as hub genes for further analyses.

**Figure 4:**
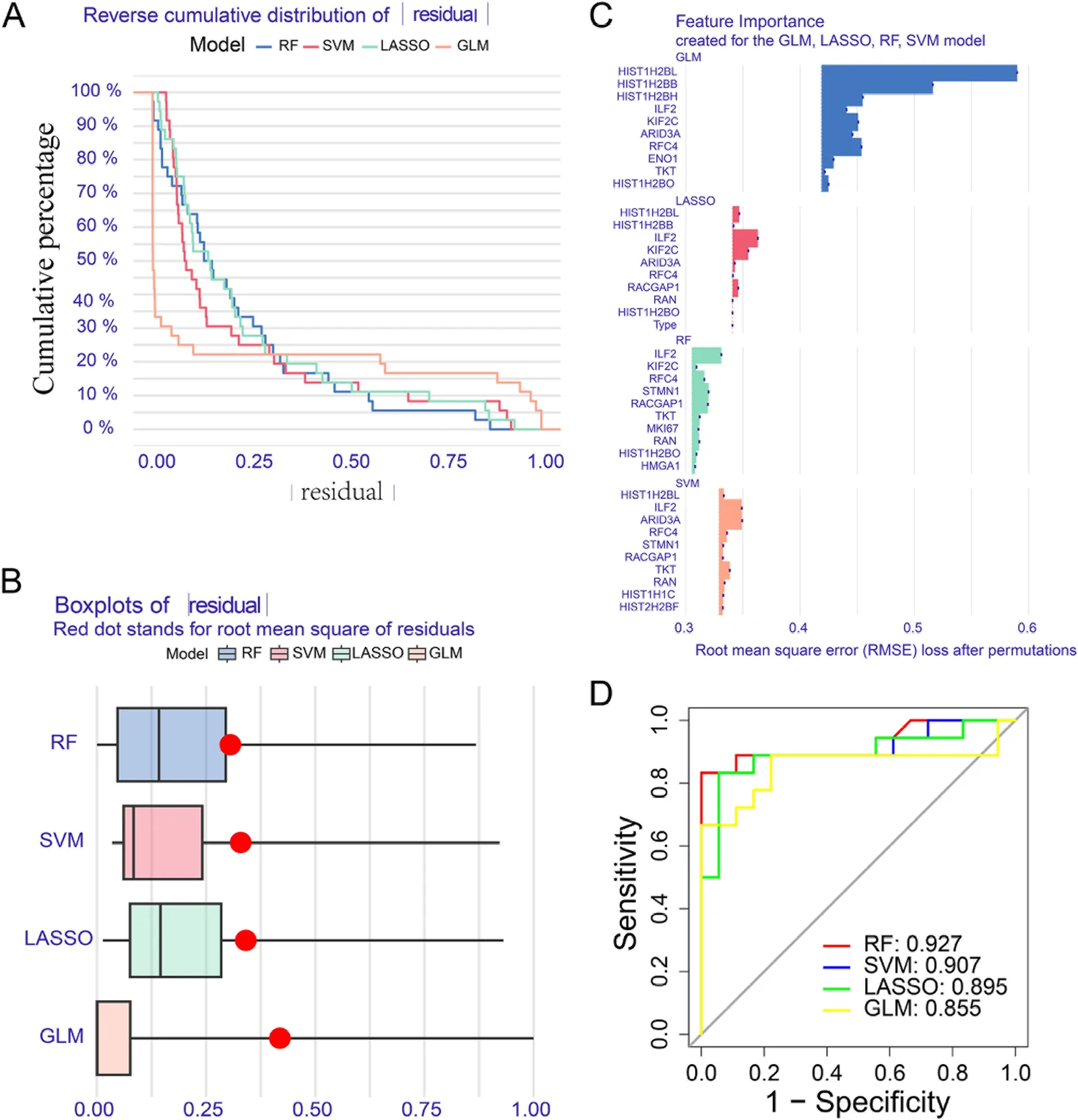
Construction and comparative evaluation of machine learning models (SVM, RF, LASSO, and GLM) for HBV-HCC prediction. (A) Cumulative distribution of residuals across all models. (B) Boxplots displayed the residuals for each machine learning model, with the red dot indicating the RMSE of the residuals. (C) Key features in the SVM, RF, LASSO, and GLM models. (D) The ROC curves of four machine learning models.

### 3.4 Expression patterns of hub genes in HBV-HCC

As illustrated in Figure 5A and B, the expression levels of TKT, RACGAP1, RFC4, STMN1, and ILF2 were notably increased in patients diagnosed with HBV-HCC according to data sourced from the GEO database. Additionally, an analysis of the TCGA database revealed that the expressions levels of TKT, RACGAP1, RFC4, STMN1, and ILF2 were significantly elevated in HCC (Figure 5C), with a marked emphasis on HBV-HCC (Figure 5D). To identify genes that may be associated with HBV infection, we conducted an analysis comparing the mRNA expression of these five genes in HBV-negative HCC cells (represented by HepG2) versus HBV-positive HCC cells (represented by HepG2.2.15). Notably, a significant increase in the expression of RACGAP1, STMN1, and ILF2 was detected in HBV-positive HCC cells, whereas TKT and RFC4 did not exhibit similar upregulation (Figure 5E). Thus, three genes (RACGAP1, STMN1, and ILF2) were considered as key hub genes for further analyses.

**Figure 5:**
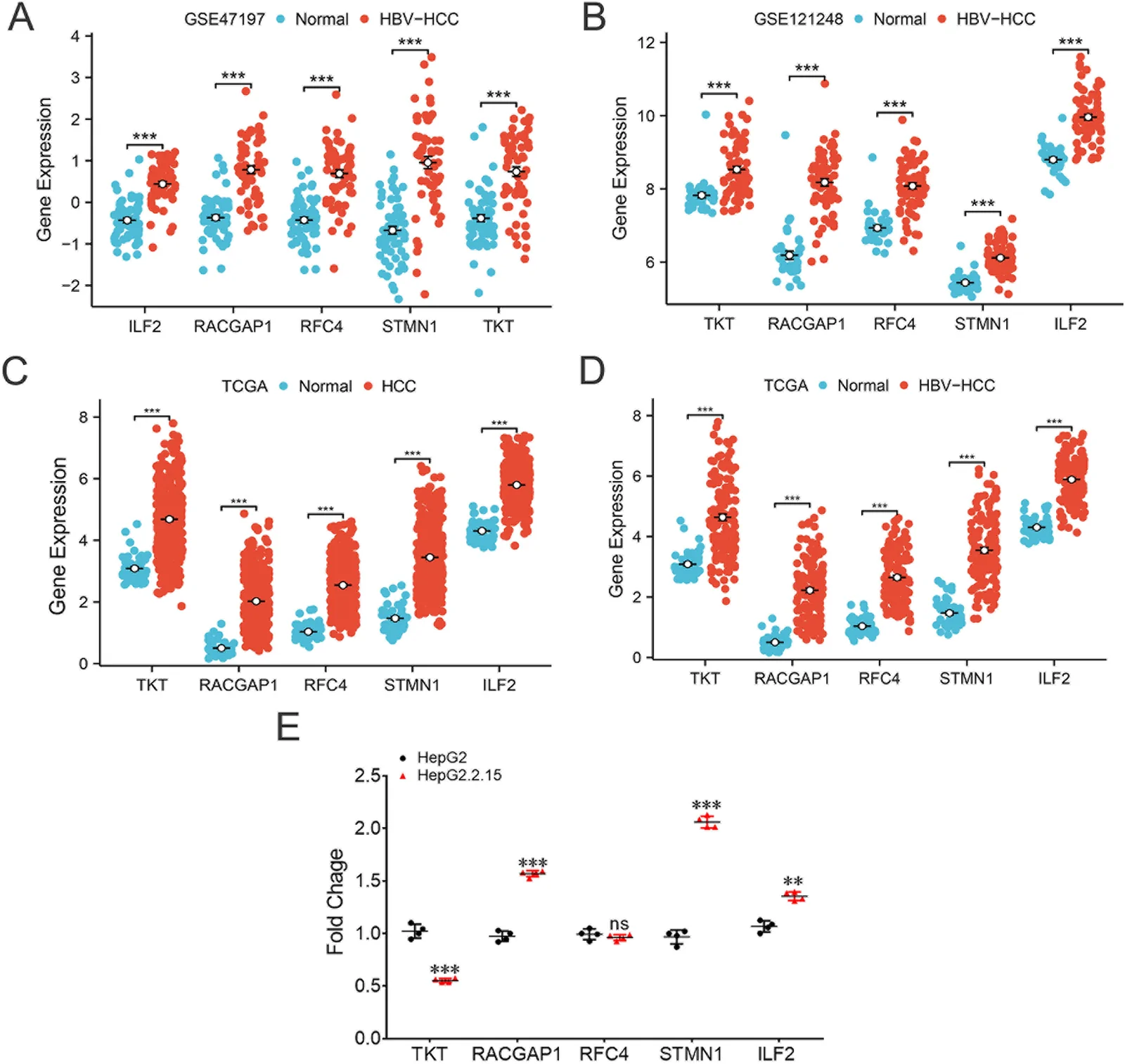
Verification of different expression levels of the five hub genes. (A) GSE47197 cohort. (B) GSE121248 cohort. (C–D) TCGA cohort. (E) qPCR. Data were shown as mean ± SD. ns: no significant, *P < 0.05, **P < 0.01, ***P < 0.001.

### 3.5 Increased expression of hub genes predicts poor prognosis in HBV-HCC

RNA-sequencing expression profiles and corresponding clinical data for 145 HBV-HCC patients were retrieved from the TCGA database. Kaplan–Meier survival analysis revealed that expression levels of RACGAP1, STMN1, and ILF2 were significantly associated with OS in HBV-HCC patients. Specifically, patients with lower expression levels of these three hub genes exhibited significantly better OS than those with higher expression levels (Figure 6A–C). Consistent findings were also observed in HCC patients sourced from TCGA (Figure 6D–F).

**Figure 6:**
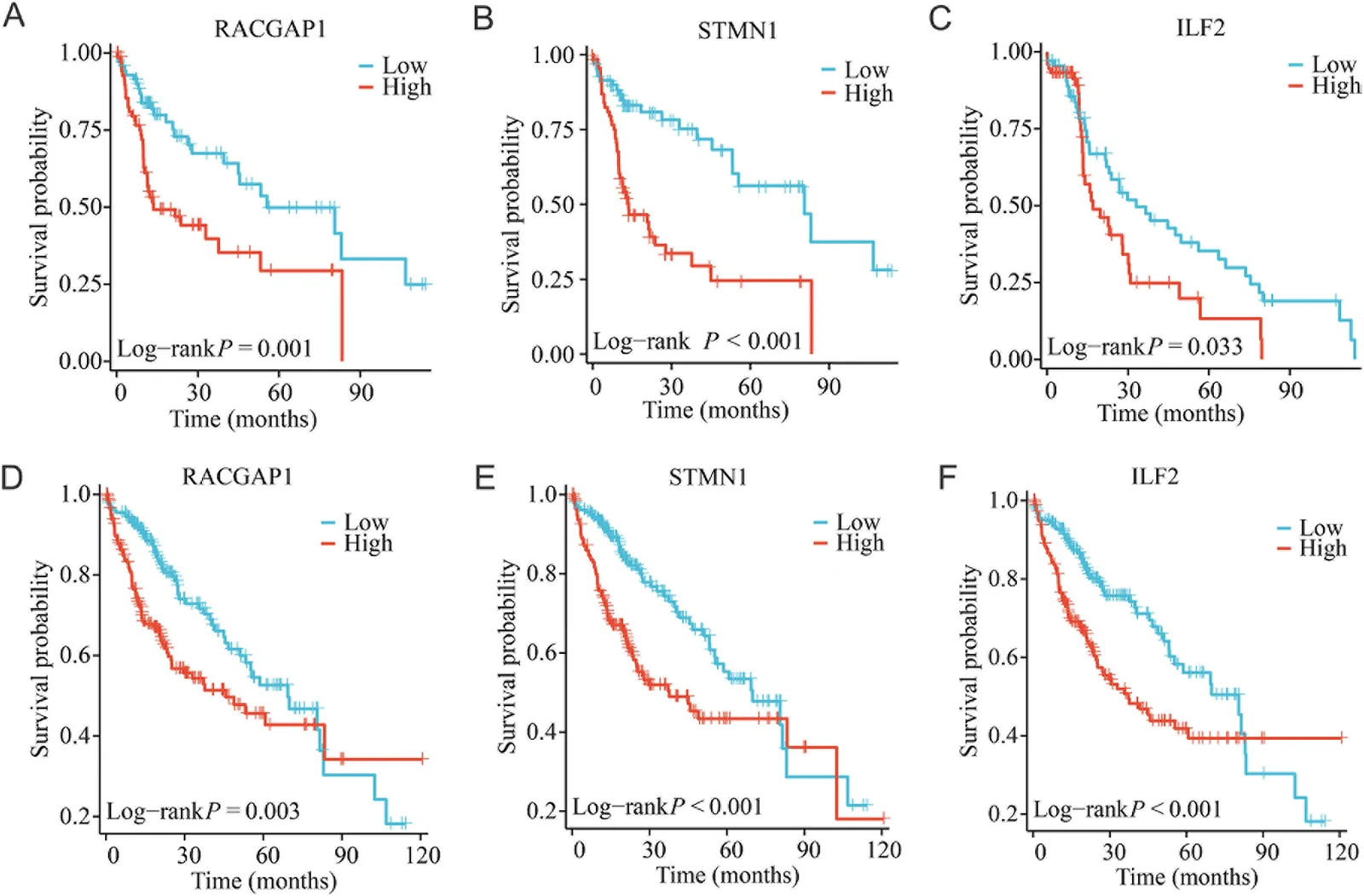
Clinical significance of key genes in TCGA cohort. (A–C) Survival analysis of RACGAP1, STMN1, and ILF2 in patients with HBV-HCC. (D–F) Survival analysis of RACGAP1, STMN1, and ILF2 in patients with HCC.

### 3.6 Construction of nomogram model

A nomogram was created in the training cohort (GSE47197) to assess how effectively the three key hub genes predict the risk of patients developing from HBV to HCC (Figure 7A). The calibration curve showed a close match between predicted and actual outcomes (Figure 7B). The DCA indicated that our model provided significant advantages for guiding clinical decisions (Figure 7C). Additionally, the nomogram’s discriminatory ability was validated using ROC analysis, yielding AUC values of 0.890 for RACGAP1, 0.885 for STMN1, and 0.905 for ILF2 (Figure 7D). Furthermore, the nomogram model demonstrated high predictive accuracy with an AUC of 0.928 (Figure 7E).

**Figure 7:**
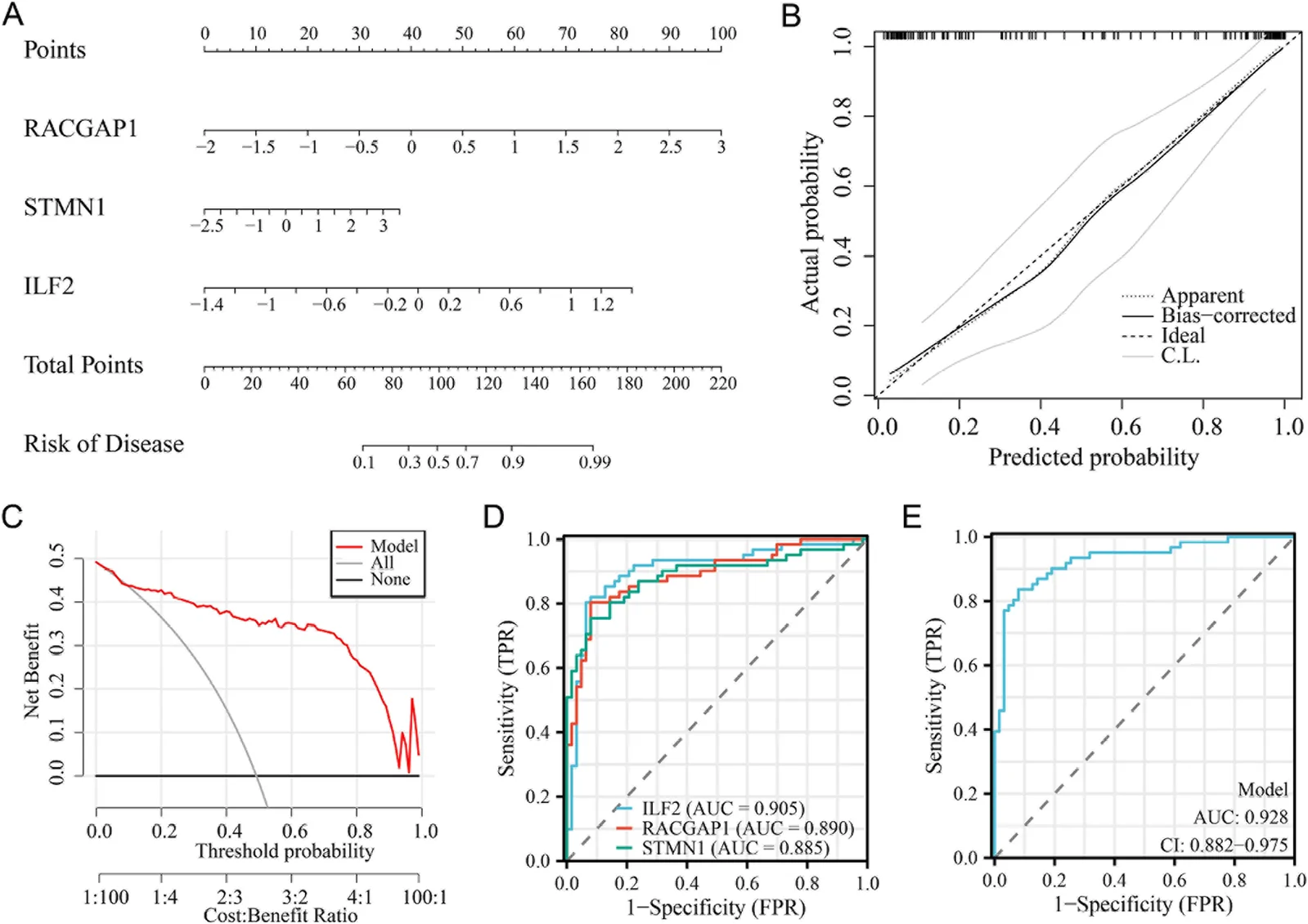
Validation of the five genes-based RF model in training cohort (GSE47197). (A) Nomogram for the predictive model. (B) Calibration curve. (C) DCA for the predictive model. (D) ROC curve of five hub genes (E) ROC curve of nomogram model.

### 3.7 Model validation

To further substantiate the five-gene model, we evaluated its reliability and reproducibility utilizing the validation dataset (GSE121248). We constructed a nomogram that integrated five predictive markers, successfully validating its outstanding predictive accuracy through the use of calibration curves, DCA, and clinical impact curves (Figure 8A–C). The ROC analysis demonstrated AUC values surpassing 0.85 for each individual predictive gene; however, the predictive model that amalgamated these genes achieved an AUC of 0.965, thus exceeding the performance of any single gene (Figure 8D and E). This suggests that the genes, RACGAP1, STMN1, and ILF2 may be reliable predictive markers for HBV-HCC.

**Figure 8:**
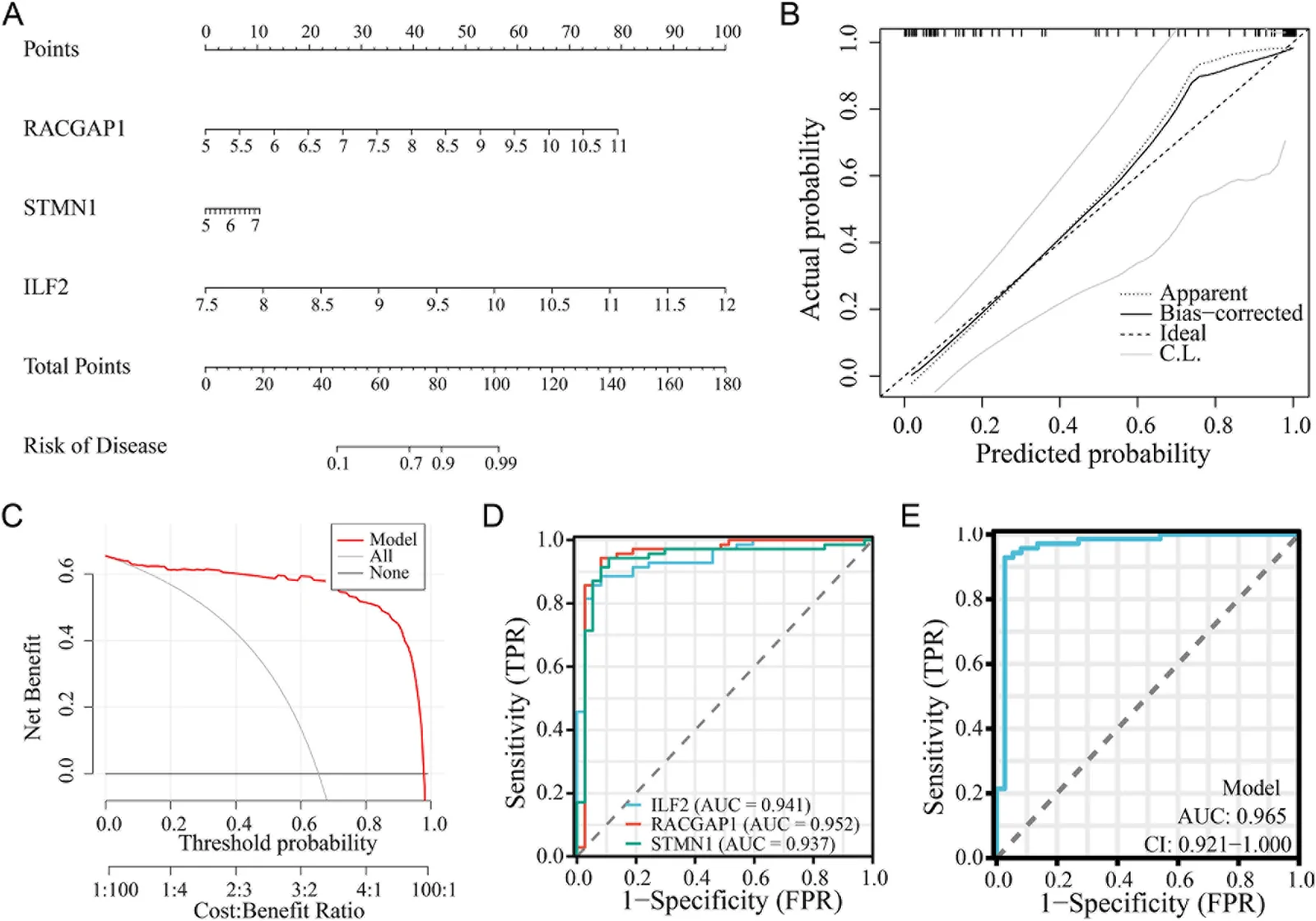
Validation of the five genes-based RF model in training cohort (GSE121248). (A) Nomogram for the predictive model. (B) Calibration curve. (C) DCA for the predictive model. (D) ROC curve of five hub genes (E) ROC curve of nomogram model.

### 3.8 Analysis of immune infiltration

A bar chart was utilized to depict the distribution of 22 distinct immune cell categories across each sample (Figure 9A). The results indicated that HBV-HCC patients had lower proportions of resting CD8 T cells, gamma delta T cells, monocytes, M1 macrophages, and activated mast cells than the HBV control groups. Conversely, they exhibited higher levels of activated CD4 T cells, M0 macrophages, and activated dendritic cells (Figure 9B). Spearson correlation analysis showed a strong positive correlation between STMN1 and RACGAP1 showed a positive correlation with activated dendritic and plasma cells, while exhibiting negative correlations with resting mast cells; while ILF2 showed a positive correlation with M0 macrophages and Tregs (Figure 9C).

**Figure 9:**
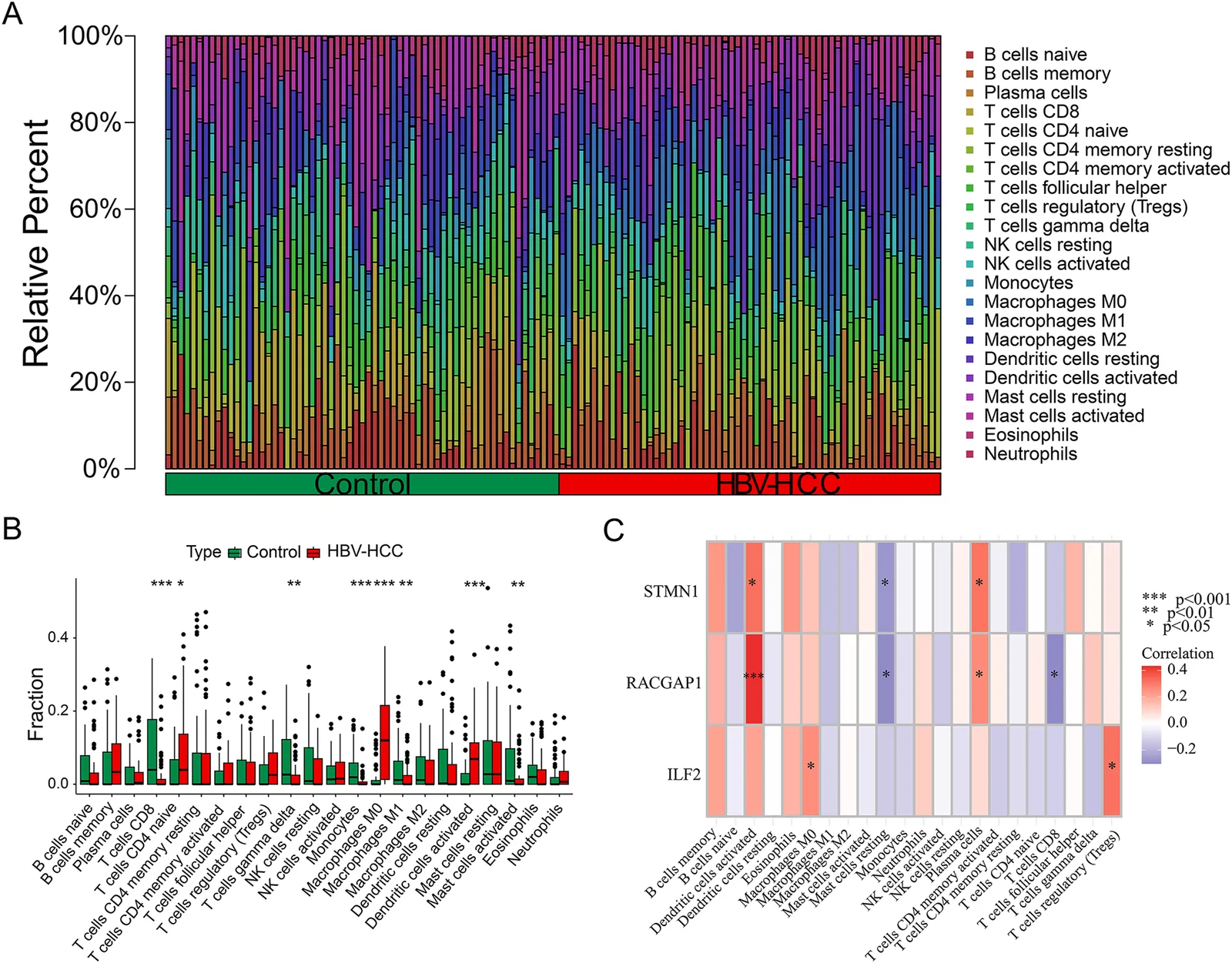
Infiltration pattern of immune cells. (A) Heatmap of the 22 immune cell proportions in each sample. (B) The different fractions of infiltrated immune cells. (C) Correlation heatmap among 22 immune cells. *P < 0.05, **P < 0.01, ***P < 0.001.

### 3.9 Identification and validation of HBV-HCC clusters

To further explore the relationship between hub genes and HBV-HCC, we performed consensus clustering analysis on 145 HBV-HCC patients from TCGA cohort. According to the relative change of the area under the CDF curve and the uniform heat map, the optimal number of clusters was determined to be four (k value = 2), which suggested two molecular patterns, and there was no significant change in the area under the CDF curve (Figure 10A–C). The 145 HBV-HCC patients can be divided into Cluster 1 (*n* = 96) and Cluster 2 (*n* = 49), and PCA effectively distinguished between the two clusters (Figure 10D). Additionally, we conducted OS analysis, showing that HBV-HCC patients in Cluster 2 exhibited worse OS than those in Cluster 1 (Figure 10E).

**Figure 10:**
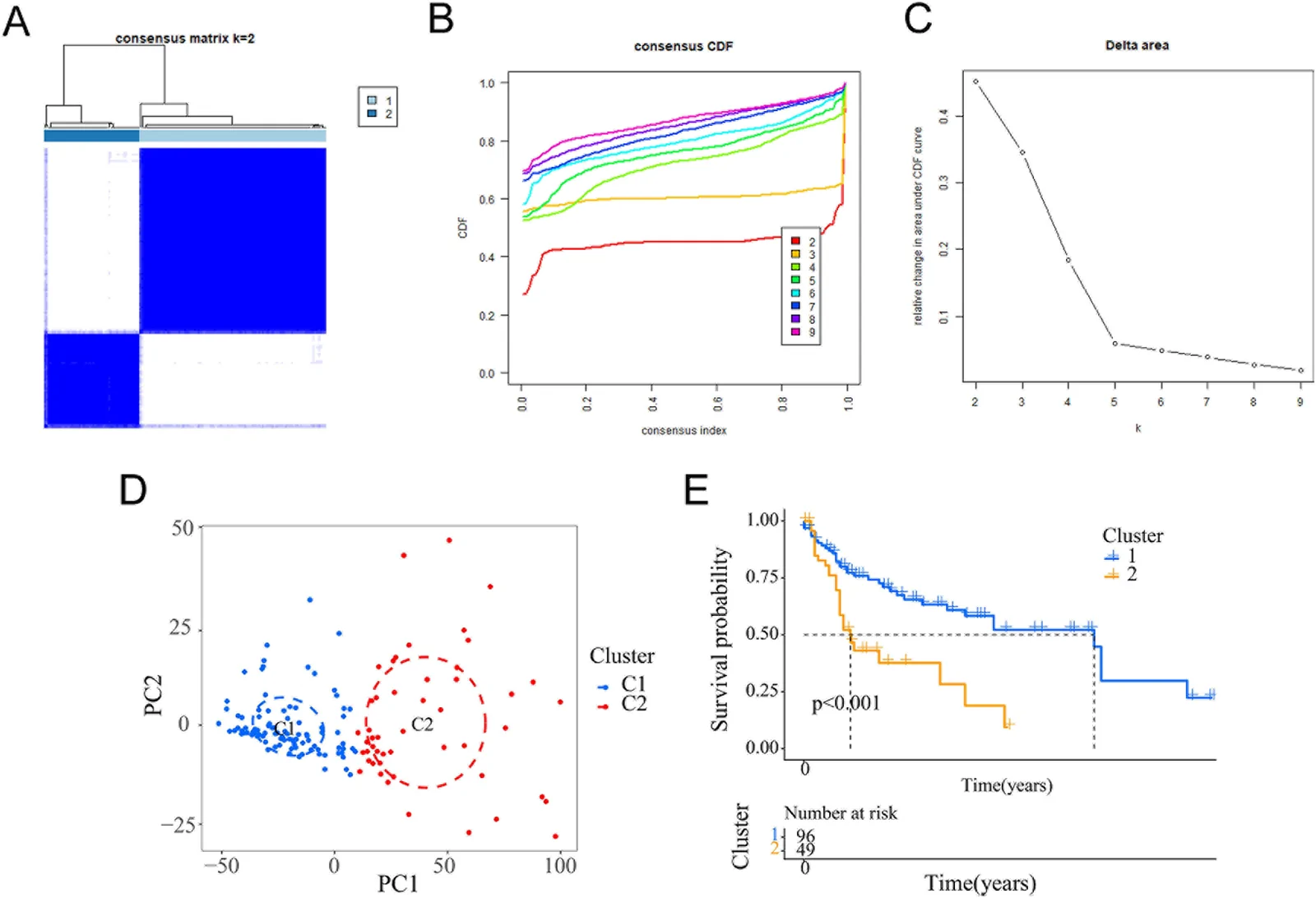
Identification of lactylation-related molecular clusters in HBV-HCC patients from TCGA cohort. (A) Consensus clustering matrix at *k* = 2. (B) The CDF curves. (C) The CDF delta area curves. (D) PCA of two distinct clusters. (E) Survival analysis of two distinct clusters.

## 4 Discussion

HCC is a highly heterogeneous tumor with poor prognosis, and chronic HBV infection is the leading cause of HBV-HCC [4], 5]. Emerging studies have revealed that LRGs participate in HCC progression, yet their clinical significance and biological function of HBV-HCC remain poorly defined. Herein, we systematically analyzed LRGs expression profiles in HBV-HCC, constructed a machine learning predictive model, and established a novel two-subtype molecular classification. Our results demonstrate that lactylation-related molecular features effectively reflect HBV-HCC heterogeneity, providing promising biomarkers and a feasible stratification strategy for clinical prognostic assessment.

Machine learning algorithms have been widely applied for tumor biomarker screening in HBV-HCC research [29]. In this study, four models (RF, SVM, LASSO, GLM) were constructed and cross-validation, and the RF model with optimal performance was selected for hub gene screening. Five candidate genes were initially identified, and further multi-cohort validation in GEO, TCGA database and HepG2.2.15 cells confirmed that three genes (RACGAP1, STMN1, ILF2) were specifically and significantly upregulated in HBV-HCC. Survival analysis further verified that high expression of these three hub genes was correlated with unfavorable overall survival, indicating their potential as novel LRG-related prognostic biomarkers for HBV-HCC.

RACGAP1 is known to drive invasive behaviours and predicts early postoperative recurrence among HBV-positive HCC patients [32]. Recent studies further revealed that RACGAP1 is preferentially up-regulated in HBV-/HCV-related HCC compared with non-viral HCC, and its expression level correlates with immune-microenvironment signatures in virus-associated HCC [33]. Consistent with previous studies, our data showed that elevated RACGAP1 was associated with poor prognosis and abnormal immune infiltration in HBV-HCC, while its linkage with lactylation still lacks experimental evidence. STMN1 modulates tumour glycolysis and promotes malignant phenotypes in human cancers [34]. Aberrant STMN1 expression is observed in HCC [35], and its elevation appears at precancerous lesions in HBV-infected cirrhotic liver [36]. Our results also confirmed its upregulation correlated with adverse prognosis in HBV-HCC. The potential crosstalk between STMN1-mediated glycolysis and lactylation requires further validation. Compared with RACGAP1 and STMN1, the biological function of ILF2 in liver malignancies remains poorly characterised. ILF2 is a multifunctional host factor capable of interacting with viral proteins and restricting replication of several RNA viruses [37], 38]. Furthermore, ILF2 cooperates with E2F1 to maintain mitochondrial homeostasis and supports tumour-promoting activities [39]. However, its exact role in HBV-HCC remains unclear. Our study found that high ILF2 expression correlated with poor prognosis and immune dysregulation in HBV-HCC. Further experiments are needed to clarify its role in HBV-related liver tumorigenesis.

In the present study, immune infiltration analysis revealed significant inverse correlations between the expression levels of these hub genes and the abundance of multiple immune cell subsets, including endothelial cells, neutrophils, B cells, cytotoxic cells, mast cells, and NK cells. Accumulating mechanistic studies have illustrated the extensive crosstalk between lactate metabolism and tumor immune suppression [40], 41]. Multiomics investigations further indicate that lactate-related gene signatures are associated with impaired anti-tumor immune infiltration and therapeutic resistance in multiple tumors [42]. For instance, lactate accumulation facilitates the recruitment of bone marrow-derived neutrophils, elevate neutrophil PD-L1 expression, and further attenuate the therapeutic efficacy of lenvatinib in HCC [43], 44]. Lactate is also capable of inducing ARG1 in macrophages and impairing the cytotoxic function of NK cells and CTLs, thereby promoting tumor immune escape [45], 46]. Collectively, our correlative results suggest an association of these hub genes with immune microenvironment characteristics in HBV-HCC, which requires further experimental verification.

Based on the expression of three core LRGs, unsupervised consensus clustering analysis successfully stratified HBV-HCC patients into two molecular subtypes with distinct prognostic outcomes. Patients in Cluster 2 exhibited poorer overall survival than those in Cluster 1. These results suggest that lactylation-related gene expression patterns can partially reflect the internal heterogeneity of HBV-HCC patients. This subgrouping approach may provide a preliminary reference for the prognostic stratification of HBV-HCC. Of note, the biological characteristics and potential clinical differences between the two subtypes remain clarified, and further investigations are also warranted.

Several limitations of this study should be acknowledged. First, all analyses were retrospectively performed based on public TCGA and GEO datasets. Further validation in prospective and multicenter clinical cohorts is needed to confirm the generalizability of our findings. Second, this study is purely bioinformatics-based. The potential associations among hub genes, lactylation modification, and tumor immune microenvironment as well as prognosis prediction remain preliminary and require further validation through *in vitro* and *in vivo* functional experiments in HBV-HCC.

## 5 Conclusions

In summary, our study demonstrated that unsupervised consensus clustering based on three key LRGs effectively stratified HBV-HCC patients into two molecular subtypes with distinct prognostic outcomes. This bioinformatic finding provides preliminary evidence that lactylation-associated molecular patterns can reflect tumor heterogeneity and serve as a potential reference for prognostic risk stratification in HBV-HCC, which may offer novel insights for individualized prognostic evaluation.

## Supplementary Material

Supplementary Material

Supplementary Material

Supplementary Material

Supplementary Material

## References

[1] Ott JJ, Stevens GA, Groeger J, Wiersma ST (2012). Global epidemiology of hepatitis B virus infection: new estimates of age-specific HBsAg seroprevalence and endemicity. Vaccine.

[2] Schweitzer A, Horn J, Mikolajczyk RT, Krause G, Ott JJ (2015). Estimations of worldwide prevalence of chronic hepatitis B virus infection: a systematic review of data published between 1965 and 2013. Lancet.

[3] El-Serag HB (2012). Epidemiology of viral hepatitis and hepatocellular carcinoma. Gastroenterology.

[4] Venook AP, Papandreou C, Furuse J, de Guevara LL (2010). The incidence and epidemiology of hepatocellular carcinoma: a global and regional perspective. Oncologist.

[5] Abdelhamed W, El-Kassas M (2024). Hepatitis B virus as a risk factor for hepatocellular carcinoma: there is still much work to do. Liver Res.

[6] Lin SY, Zhang A, Lian J, Wang J, Chang TT, Lin YJ (2021). Recurrent HBV integration targets as potential drivers in hepatocellular carcinoma. Cells.

[7] An P, Xu J, Yu Y, Winkler CA (2018). Host and viral genetic variation in HBV-related hepatocellular carcinoma. Front Genet.

[8] Han YF, Zhao J, Ma LY, Yin JH, Chang WJ, Zhang HW (2011). Factors predicting occurrence and prognosis of hepatitis-B-virus-related hepatocellular carcinoma. World J Gastroenterol.

[9] Liu Y, Veeraraghavan V, Pinkerton M, Fu J, Douglas MW, George J (2021). Viral biomarkers for hepatitis B virus-related hepatocellular carcinoma occurrence and recurrence. Front Microbiol.

[10] Chen AN, Luo Y, Yang YH, Fu JT, Geng XM, Shi JP (2021). Lactylation, a novel metabolic reprogramming code: current status and prospects. Front Immunol.

[11] Peng X, He Z, Yuan D, Liu Z, Rong P (2024). Lactic acid: the culprit behind the immunosuppressive microenvironment in hepatocellular carcinoma. Biochim Biophys Acta Rev Cancer.

[12] Zhang D, Tang Z, Huang H, Zhou G, Cui C, Weng Y (2019). Metabolic regulation of gene expression by histone lactylation. Nature.

[13] Xu Y, Hao X, Ren Y, Xu Q, Liu X, Song S (2022). Research progress of abnormal lactate metabolism and lactate modification in immunotherapy of hepatocellular carcinoma. Front Oncol.

[14] Feng F, Wu J, Chi Q, Wang S, Liu W, Yang L (2024). Lactylome analysis unveils lactylation-dependent mechanisms of stemness remodeling in the liver cancer stem cells. Adv Sci (Weinh).

[15] Zhao P, Qiao C, Wang J, Zhou Y, Zhang C (2024). Histone lactylation facilitates hepatocellular carcinoma progression by upregulating endothelial cell-specific molecule 1 expression. Mol Carcinog.

[16] Lu Y, Zhu J, Zhang Y, Li W, Xiong Y, Fan Y (2024). Lactylation-driven IGF2BP3-mediated serine metabolism reprogramming and RNA m6A-modification promotes lenvatinib resistance in HCC. Adv Sci (Weinh).

[17] Yu Y, Li Y, Zhou L, Cheng X, Gong Z (2024). Hepatic stellate cells promote hepatocellular carcinoma development by regulating histone lactylation: novel insights from single-cell RNA sequencing and spatial transcriptomics analyses. Cancer Lett.

[18] Cai D, Yuan X, Cai DQ, Li A, Yang S, Yang W (2023). Integrative analysis of lactylation-related genes and establishment of a novel prognostic signature for hepatocellular carcinoma. J Cancer Res Clin Oncol.

[19] Cheng Z, Huang H, Li M, Liang X, Tan Y, Chen Y (2023). Lactylation-related gene signature effectively predicts prognosis and treatment responsiveness in hepatocellular carcinoma. Pharmaceuticals.

[20] Yang B, Bao W, Zhang Y, Liu Q (2019). RNDEtree: regulatory network with differential equation based on flexible neural tree with novel criterion function. IEEE Access.

[21] Bao W, Yang B (2023). Protein acetylation sites with complex-valued polynomial model. Front Comput Sci.

[22] Bao W, Yang B (2025). TAPE_selection: Organelle proteins classification with TAPE feature selection. IEEE ACM Trans Comput Biol Bioinf.

[23] Yang B, Bao W (2019). Complex-valued ordinary differential equation modeling for time series identification. IEEE Access.

[24] Li W, Liu Y, Liu W, Tang ZR, Dong S, Li W (2022). Machine learning-based prediction of lymph node metastasis among osteosarcoma patients. Front Oncol.

[25] Montesinos López OA, Montesinos López A, Crossa J (2022). Support vector machines and support vector regression. Multivariate statistical machine learning methods for genomic prediction.

[26] Rabe-Hesketh S, Skrondal A (2010). Generalized linear mixed models. International encyclopedia of education.

[27] Chatterjee T, Chowdhury R (2017). Improved sparse approximation models for stochastic computations. Handbook of neural computation.

[28] Yin P, Mao N, Zhao C, Wu J, Sun C, Chen L (2019). Comparison of radiomics machine-learning classifiers and feature selection for differentiation of sacral chordoma and sacral giant cell tumour based on 3D computed tomography features. Eur Radiol.

[29] Li J, Huang L, He B, Xie H, Wu Q, Chen L (2025). Identification of lysine crotonylation-driven molecular clusters and immune dysregulation in HBV-related hepatocellular carcinoma via bioinformatics and machine learning. Transl Cancer Res.

[30] Iasonos A, Schrag D, Raj GV, Panageas KS (2008). How to build and interpret a nomogram for cancer prognosis. J Clin Oncol.

[31] Newman AM, Liu CL, Green MR, Gentles AJ, Feng W, Xu Y (2015). Robust enumeration of cell subsets from tissue expression profiles. Nat Methods.

[32] Wang SM, Ooi LL, Hui KM (2011). Upregulation of Rac GTPase-activating protein 1 is significantly associated with the early recurrence of human hepatocellular carcinoma. Clin Cancer Res.

[33] Saeed MM, Ma X, Fu X, Ullah I, Ali T, Bai C (2025). RACGAP1 and MKI67 are potential prognostic biomarker in hepatocellular carcinoma caused by HBV/HCV via lactylation. Front Oncol.

[34] Wang S, Su T, Tong H, Zhou D, Ma F, Ding J (2021). Circβ-catenin promotes tumor growth and Warburg effect of gallbladder cancer by regulating STMN1 expression. Cell Death Discov.

[35] Cai Y, Fu Y, Liu C, Wang X, You P, Li X (2022). Stathmin 1 is a biomarker for diagnosis of microvascular invasion to predict prognosis of early hepatocellular carcinoma. Cell Death Dis.

[36] Ahn EY, Yoo JE, Rhee H, Kim MS, Choi J, Ko JE (2014). Increased expression of stathmin and elongation factor 1α in precancerous nodules with telomere dysfunction in hepatitis B viral cirrhotic patients. J Transl Med.

[37] Wen X, Bian T, Zhang Z, Zhou L, Ge X, Han J (2017). Interleukin-2 enhancer binding factor 2 interacts with the nsp9 or nsp2 of porcine reproductive and respiratory syndrome virus and exerts negatively regulatory effect on the viral replication. Virol J.

[38] Cui X, Qian P, Rao T, Wei Y, Zhao F, Zhang H (2019). Cellular interleukin enhancer-binding factor 2, ILF2, inhibits Japanese encephalitis virus replication in vitro. Viruses.

[39] Zhao M, Liu Y, Chang J, Qi J, Liu R, Hou Y (2019). ILF2 cooperates with E2F1 to maintain mitochondrial homeostasis and promote small cell lung cancer progression. Cancer Biol Med.

[40] Sun K, Shen Y, Xiao X, Xu H, Zhang Q, Li M (2024). Crosstalk between lactate and tumor-associated immune cells: clinical relevance and insight. Front Oncol.

[41] Wang X, Xiong D, Cui S, Duan B, Ding G, Huang Y (2026). Lactate signaling and immune suppression in tumors: mechanisms and therapeutic implications. Cell Oncol.

[42] Feng B, Zhu Y, Zhang Z, Wang Y, Schuler PJ, Hess J (2026). Tumor lactate metabolism shapes immune suppression and therapeutic resistance revealed by integrative multi-omics and digital pathology. Front Immunol.

[43] Khatib-Massalha E, Bhattacharya S, Massalha H, Biram A, Golan K, Kollet O (2020). Lactate released by inflammatory bone marrow neutrophils induces their mobilization via endothelial GPR81 signaling. Nat Commun.

[44] Deng H, Kan A, Lyu N, He M, Huang X, Qiao S (2021). Tumor-derived lactate inhibit the efficacy of lenvatinib through regulating PD-L1 expression on neutrophil in hepatocellular carcinoma. J Immunother Cancer.

[45] Ohashi T, Akazawa T, Aoki M, Kuze B, Mizuta K, Ito Y (2013). Dichloroacetate improves immune dysfunction caused by tumor-secreted lactic acid and increases antitumor immunoreactivity. Int J Cancer.

[46] Ding J, Karp JE, Emadi A (2017). Elevated lactate dehydrogenase (LDH) can be a marker of immune suppression in cancer: interplay between hematologic and solid neoplastic clones and their microenvironments. Cancer Biomark.

