# Supplementary material for: Characterization of lactylation-related genes for predictive model development and molecular subtyping in HBV-associated hepatocellular carcinoma
Source: Open Life Sci. 2026 Nov 6;21(1):20251394. doi: 10.1515/biol-2025-1394 (PMC13741892; doi:10.1515/biol-2025-1394)
Supplement: Supplementary file 2 [file j_biol-2025-1394_suppl_002.docx]

|  | **Supplementary Table S1:** Difference expression datasets referenced in this study | | | | | | |
| --- | --- | --- | --- | --- | --- | --- | --- |
| GSE dateset | | Organism | Sample number | Experiment type | HBV status | Platform |  |
| GSE47197 | | Homo sapiens | Adjacent normal liver tissues: 63  HBV-HCC: 61 | Expression profiling by array | Positive | GPL16699 |  |
| GSE121248 | | Homo sapiens | Adjacent normal liver tissues: 37  HBV-HCC: 70 | Expression profiling by array | Positive | GPL570 |  |
