# Supplementary material for: Characterization of lactylation-related genes for predictive model development and molecular subtyping in HBV-associated hepatocellular carcinoma
Source: Open Life Sci. 2026 Nov 6;21(1):20251394. doi: 10.1515/biol-2025-1394 (PMC13741892; doi:10.1515/biol-2025-1394)
Supplement: Supplementary file 3 [file j_biol-2025-1394_suppl_003.docx]

**Supplementary Table S2:** Supplementary Table S2. A total of 332 Lactylation-related genes (LRGs) were obtained from the MsigDB

Gene symbol

ACAT2

ACIN1

ADAR

ADNP

AHNAK

ALB

ALDH1A1

ALDOA

ALDOB

ALYREF

ARGLU1

ARID1A

ARID3A

ARID3B

ARPP19

BCLAF1

BOLA2

BRD4

BTF3

BZW1

BZW2

C19orf53

CACYBP

CALD1

CALM1

CALML5

CALR

CBR1

CBX3

CBX5

CCNA2

CCT5

CD2BP2

CDC5L

CDV3

CDV3

CDYL

CEBPZ

CFDP1

CHD4

CHERP

CNN2

CNN3

COPS4

CRABP2

CSRP1

CSRP2

CWC15

DDX17

DDX18

DDX21

DDX39A

DDX39B

DDX3X

DDX41

DDX42

DDX46

DDX5

DECR1

DFFA

DHRS7

DHX16

DHX9

EAF1

ECHDC1

EDF1

EEF1A1

EEF1G

EEF2

EHMT2

EIF3D

EIF3J

EIF4G1

EIF4G2

EIF4H

EMG1

ENO1

ENSA

FABP5

FAM50A

FKBP3

FLYWCH2

FUBP1

G6PD

GAPDH

GATAD2A

GATAD2B

GFAP

GIGYF2

GTF2F1

GTF2I

H1-2

H1-3

H1-5

H2AFV

H2AFZ

H2AJ

H2AX

H2AZ1

H2AZ2

H2BC13

H2BC14

H2BC18

H2BC5

H2BU1

H3-3A

H3C1

H3C15

H4C1

HCFC1

HDAC1

HDAC2

HDGF

HDGFL2

HEXIM1

HIST1H1C

HIST1H2BB

HIST1H2BD

HIST1H2BH

HIST1H2BK

HIST1H2BL

HIST1H2BN

HIST1H2BO

HIST1H3A

HIST1H4A

HIST2H2BE

HIST2H2BF

HISTIH2BH

HLTF

HMGA1

HMGB1

HMGN1

HMGN2

HMGN3

HMGN4

HNRNPA1

HNRNPC

HNRNPD

HNRNPF

HNRNPH1

HNRNPK

HNRNPL

HNRNPM

HNRNPU

HSDL2

HSPE1

IARS2

IFI16

IK

IKZF1

ILF2

ILF3

IRF2BP2

JMJD1C

JPT1

JPT2

KHDRBS1

KHSRP

KIF2C

KRT1

KRT10

LAP3

LCP1

LDHB

LEMD3

LGALS1

LRPPRC

LSP1

MAGOH

MAGOHB

MAP2K4

MAPRE1

MBD2

MBP

MDC1

MKI67

MNDA

MPHOSPH6

MSN

MTA1

MTA2

MYH13

NCDN

NCL

NEFL

NHLRC2

NOC3L

NOLC1

NONO

NOP2

NPM1

NSUN2

NUCKS1

NUDT21

NUDT5

NUP133

NUP50

PABPC1

PABPN1

PAK2

PARP1

PCBP1

PCBP2

PCMT1

PCNP

PDAP1

PDLIM1

PES1

PFKM

PFKP

PFN1

PGK1

PHC3

PHF6

PKM2

POLDIP3

PPIA

PPIL4

PPM1G

PPP1CB

PPP1CC

PPP1R2B

PRAM1

PRCC

PRDX1

PRKDC

PRPF6

PSMA7

PSMC1

PSME3IP1

PTBP1

PTMA

RACGAP1

RALYL

RAN

RANBP2

RB1

RBM10

RBM14

RBM17

RBM25

RBM39

RBMX

RCC2

RECQL

RFC1

RFC4

RIMS1

RPA1

RPL13

RPL14

RPL22

RPL24

RPL29

RPL5

RPS11

RPS23

RPS27A

RRP1B

RSL1D1

S100A11

S100A4

S100A6

SAFB

SARNP

SATB1

SET

SF3A1

SF3B1

SFPQ

SFPQ

SH3GL1

SMAP

SMARCA5

SMARCC1

SMARCC2

SMC3

SNRPA1

SOD1

SPR

SPR14

SRP14

SRRM1

SRRM2

SSB

STMN1

SUB1

SUMO2

TCOF1

TERF2

THOC2

THRAP3

THUMPD1

TKT

TMA7

TMPO

TMSB4X

TOP2B

TP53

TPM4

TPR

TRIM28

TRIR

TSSC4

U2AF2

U2SURP

UBE2E1

UBE2M

UPF1

VARS

VIM

WAS

WBP11

WDR33

WIZ

XPO5

XRCC4

YLPM1

ZC3H18

ZC3H4

ZMYM3

ZNF207

ZNF280C

ZNF706

ZNFX1

ZRANB2

ZYX

EP300

HDAC3

SIRT1

SIRT2

SIRT3
