# Supplementary material for: Characterization of lactylation-related genes for predictive model development and molecular subtyping in HBV-associated hepatocellular carcinoma
Source: Open Life Sci. 2026 Nov 6;21(1):20251394. doi: 10.1515/biol-2025-1394 (PMC13741892; doi:10.1515/biol-2025-1394)
Supplement: Supplementary file 4 [file j_biol-2025-1394_suppl_004.docx]

| **Supplementary Table S3:** The primer sequences of hub genes | | |
| --- | --- | --- |
| **Gene** | **Forward primer (5′-3′)** | **Reverse primer (5′-3′)** |
| TKT | CCAAGTGATGGCGTTGCTACAG | TTGTCCGACCTGGAAGTCCTCA |
| RFC4 | GGCAGCTTTAAGACGTACCATGG | TCTGACAGAGGCTTGAAGCGGA |
| RACGAP1 | ATGCTGGCAGACTTTGTGTCCC | CAGCCAGAGATCCTATACAGGC |
| STMN1 | AGAACCGAGAGGCACAAATGGC | TCTCGTCAGCAGGGTCTTTGGA |
| ILF2 | AGGACTGTTCCTGCCAGGTTCA | GAGGATTCGGACGAGAGTCTGA |
| GAPDH | GTCTCCTCTGACTTCAACAGCG | ACCACCCTGTTGCTGTAGCCAA |
